# Supplementary material for: Fast and pervasive diagenetic isotope exchange in foraminifera tests is species-dependent
Source: Nat Commun. 2022 Jan 10;13:113. doi: 10.1038/s41467-021-27782-8 (PMC8748890; doi:10.1038/s41467-021-27782-8)
Supplement: Supplementary file 1 — Supplementary Information [file 41467_2021_27782_MOESM1_ESM.pdf]

Supplementary Information for:

Fast and pervasive diagenetic isotope exchange in foraminifera tests is species-dependent

Deyanira Cisneros-Lazaro[1*], Arthur Adams[1], Jinming Guo[1], Sylvain Bernard[2], Lukas P. Baumgartner[3], Damien Daval[4], Alain Baronnet[5], Olivier Grauby[5], Torsten Vennemann[6], Jarosław Stolarski[7], Stéphane Escrig[1], Anders Meibom[1,3*]

* deyanira.cisneroslazaro@epfl.ch, +41779494386, anders.meibom@epfl.ch +41216938014; EPFL ENAC IIE LGB, GR C2 524 (GR building), Station 2, CH-1015 Lausanne

This PDF includes:

Supplementary Fig. 1: High magnification SEM images of the sub-spherical calcite particles.

Supplementary Fig. 2: Stereo microscope images of *Ammonia sp.* tests.

Supplementary Fig. 3: Stereo microscope images of *H. Germanica* tests.

Supplementary Fig. 4: Stereo microscope images of *A. Lessonii* tests.

Supplementary Fig. 5: High magnification SEM images of *Ammonia sp.* test surfaces.

Supplementary Fig. 6: High magnification SEM images of *H. Germanica.* test surfaces.

Supplementary Fig. 7: High magnification SEM images of *A. Lessonii* test surfaces.

Supplementary Table 1: Summary table of bulk oxygen isotope measurements of foraminifera tests.

Supplementary Fig. 8: NanoSIMS images of *Ammonia sp., A. lessonii* and *H. germanica* tests after 6 day simulated diagenesis treatment.

Supplementary Fig. 9: NanoSIMS images of an *Ammonia sp.* test after 4 hour simulated diagenesis treatment.

Supplementary Fig. 10: NanoSIMS images of an Iceland spar crystal and foraminifera tests after 6 day simulated diagenesis treatment.

Supplementary Fig. 11: The 4 key steps for quantifying cogwheel structures.

Supplementary Table 2 Cogwheel interface density quantification parameters.

Supplementary Table 3: Parameters used to calculate the diagenesis resistance ratio.

Supplementary Fig. 12: SEM image of the wall texture of benthic foraminifera *Cibicidoides havanensis* from DSDP Site 522 (Walvis Ridge).

Supplementary References

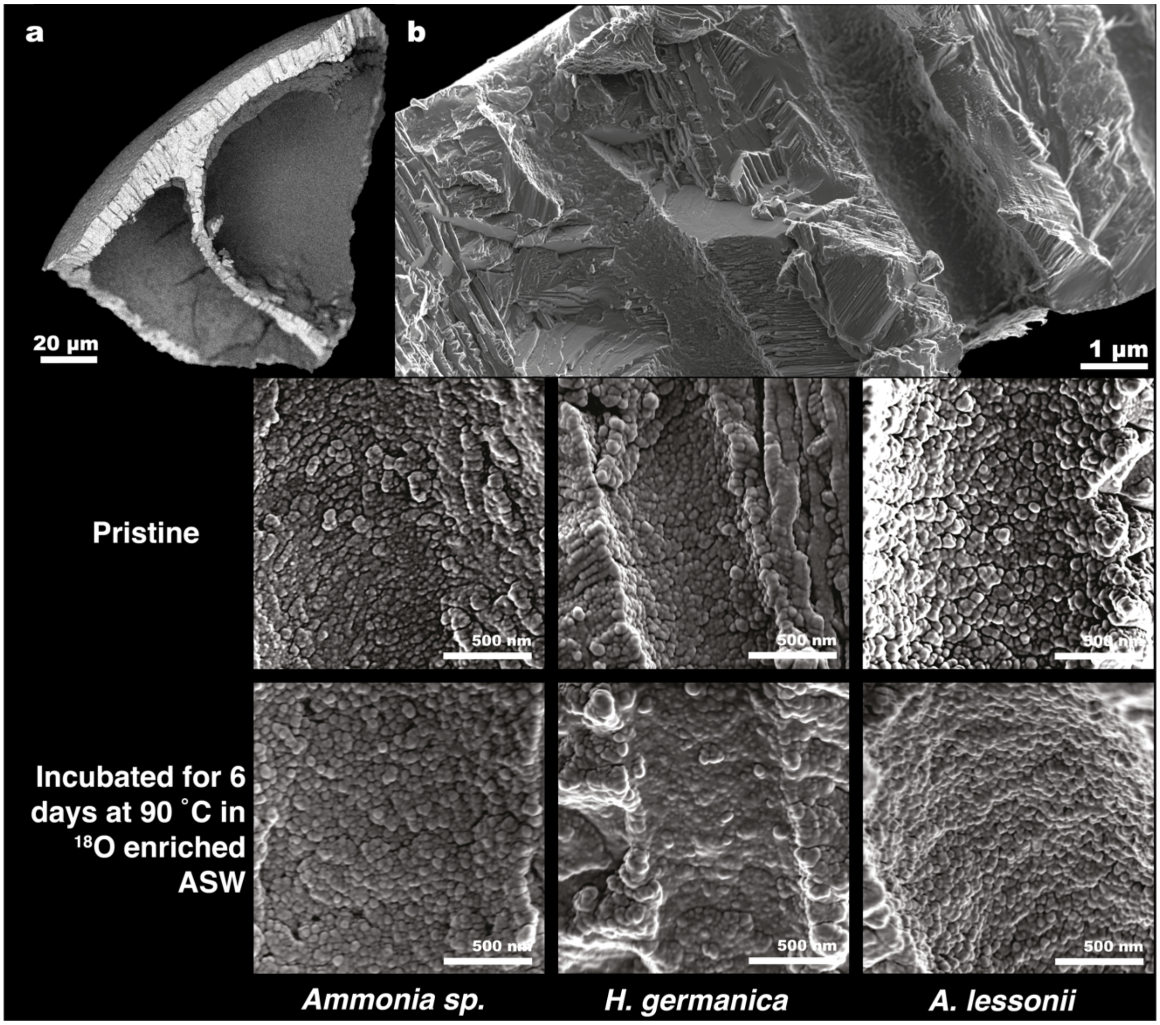

Supplementary Fig. 1 High magnification (35 000×) SEM images of the sub-spherical calcite particles that make up the test wall of three species of foraminifera, as seen on the surfaces of broken test chambers (a and b). There are no clear differences in the texture of the nanoparticles in pristine foraminifera tests of *Ammonia sp., H. germanica* and *A. lessonii* compared to tests that were incubated for 6 days at 90 °C in a seawater analogue with a $^{18}O/^{16}O$ ratio of 0.30. Notice that all high magnification images were taken at the same scale.

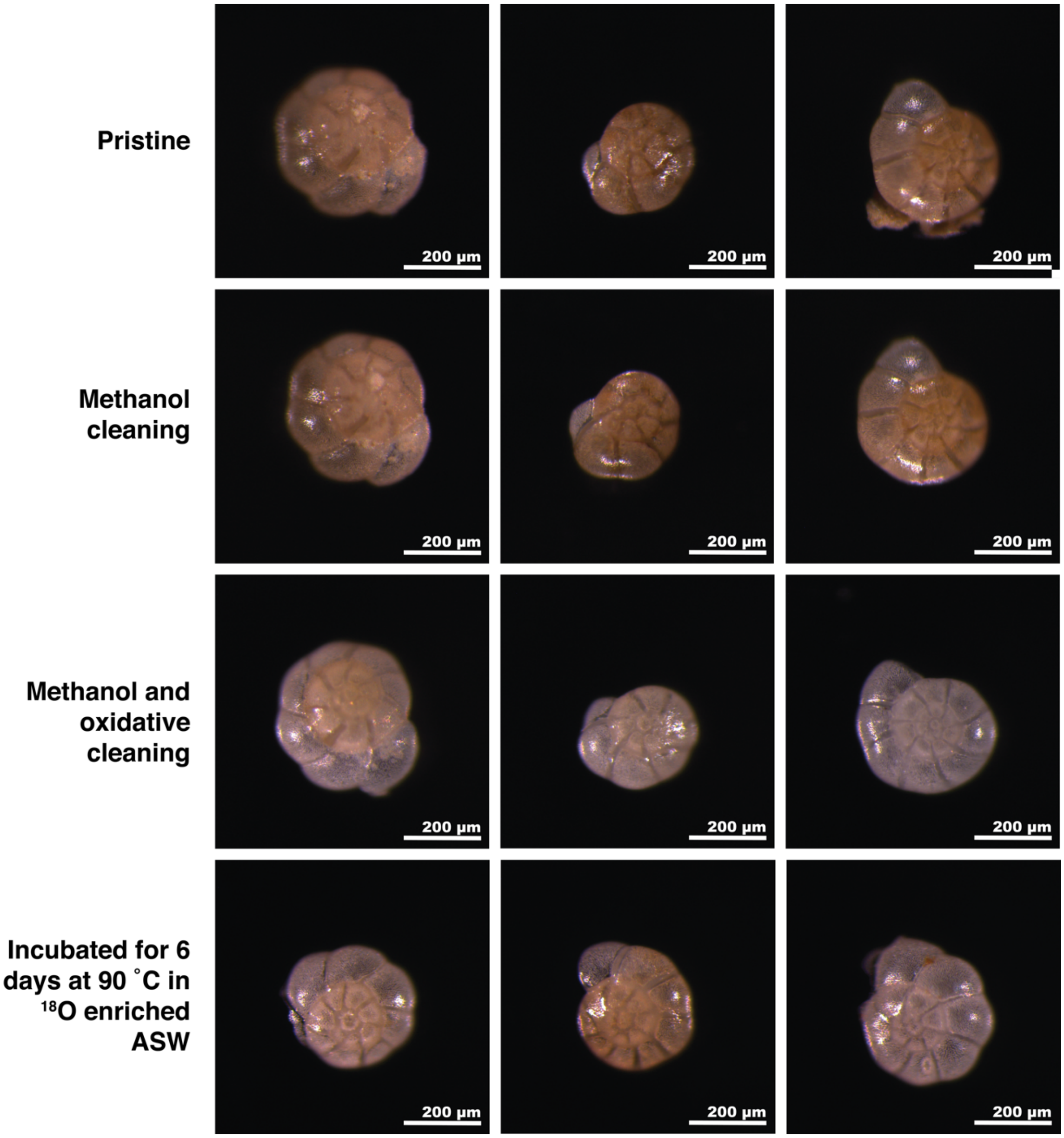

Supplementary Fig. 2 Stereo microscope images of *Ammonia sp.* tests that are pristine (1st row), cleaned only with methanol (2nd row), cleaned with methanol and an oxidative treatment (3rd row) and tests that were incubated for 6 days at 90 °C in artificial seawater (ASW) with a $^{18}O/^{16}O$ ratio of 0.30 after methanol cleaning and an oxidative treatment.

All images were taken at the same scale, under identical illumination conditions, and with the same microscope and camera.

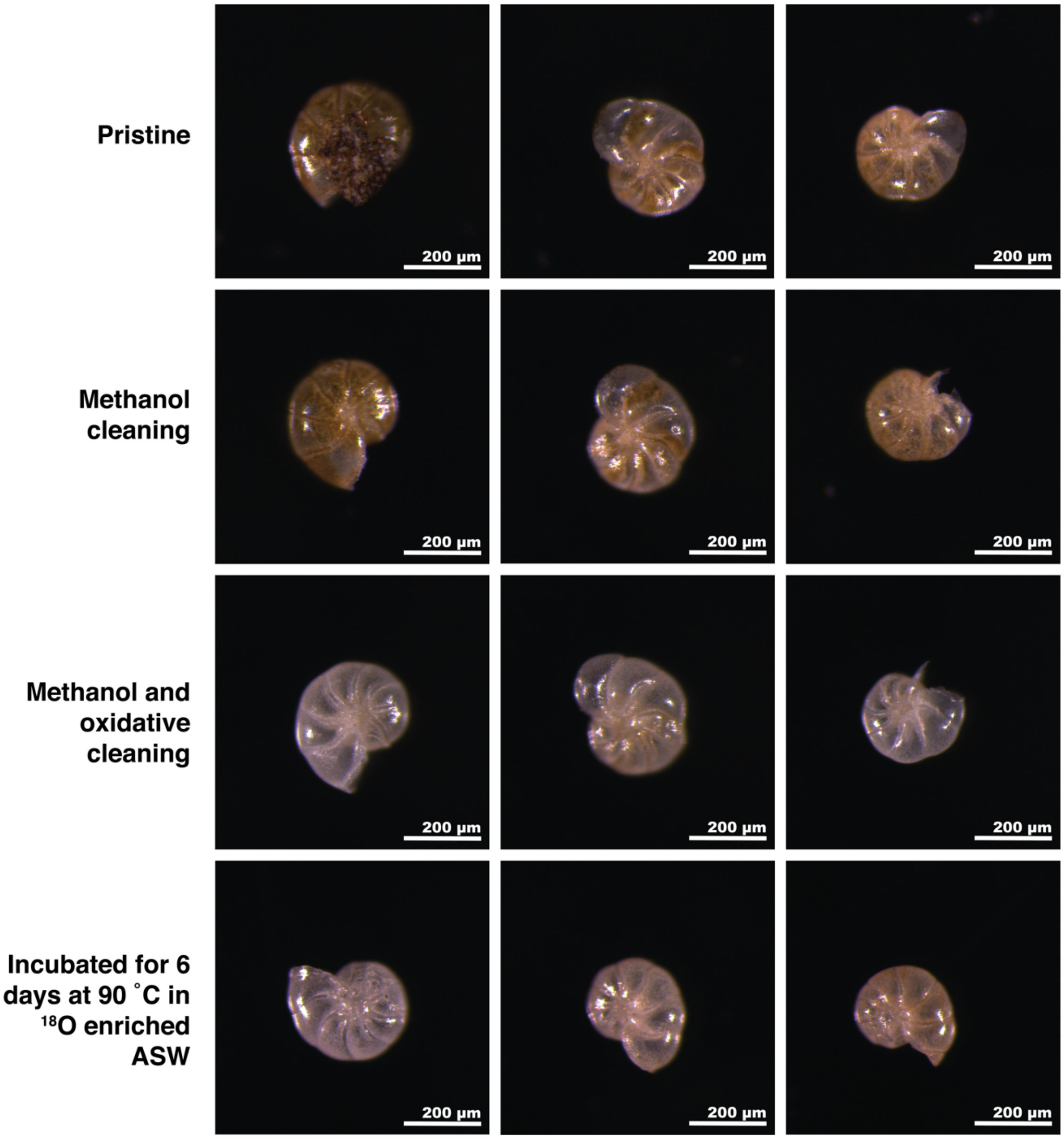

Supplementary Fig. 3 Optical images of *H. germanica.* tests that are pristine (1st row), cleaned only with methanol (2nd row), cleaned with methanol and an oxidative treatment (3rd row) and tests that were incubated for 6 days at 90 °C in artificial seawater (ASW) with a $^{18}O/^{16}O$ ratio of 0.30 after methanol cleaning and an oxidative treatment. All

images were taken at the same scale, under identical illumination conditions, and with the same microscope and camera.

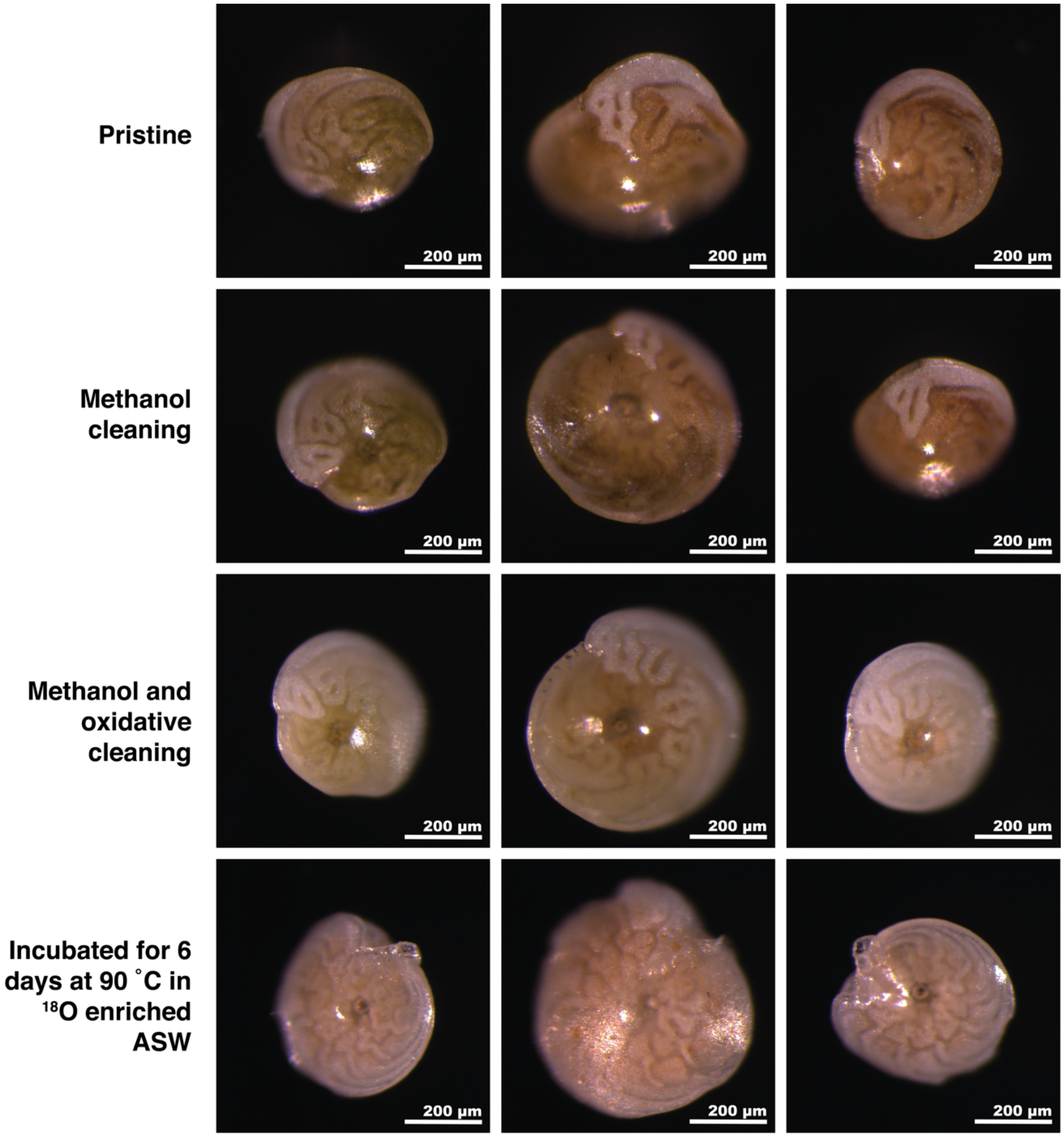

Supplementary Fig. 4 Optical images of *A. lessonii.* tests that are pristine (1st row), cleaned only with methanol (2nd row), cleaned with methanol and an oxidative treatment (3rd row) and tests that were incubated for 6 days at 90 °C in artificial seawater (ASW) with a $^{18}O/^{16}O$ ratio of 0.30 after methanol cleaning and an oxidative treatment. All

images were taken at the same scale, under identical illumination conditions, and with the same microscope and camera.

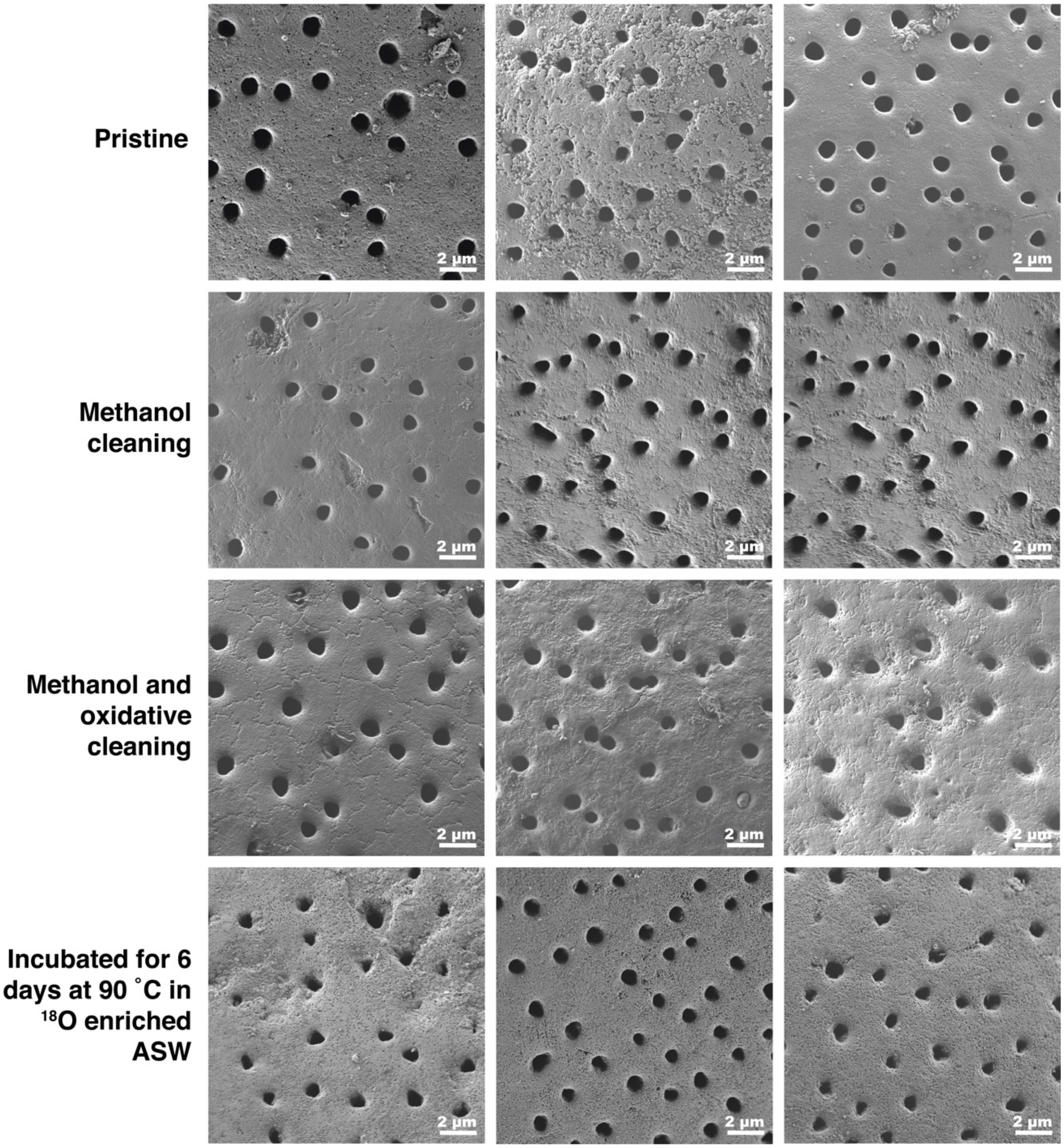

Supplementary Fig. 5 High magnification (5000×) SEM images of the surfaces of the penultimate chamber of *Ammonia sp.* comparing tests that are pristine (1st row), cleaned only with methanol (2nd row), cleaned with methanol and an oxidative treatment (3rd row) to tests that were incubated for 6 days at 90 °C in artificial seawater (ASW) with a

$^{18}O/^{16}O$ ratio of 0.30 after methanol cleaning and an oxidative treatment. All images were taken at the same scale.

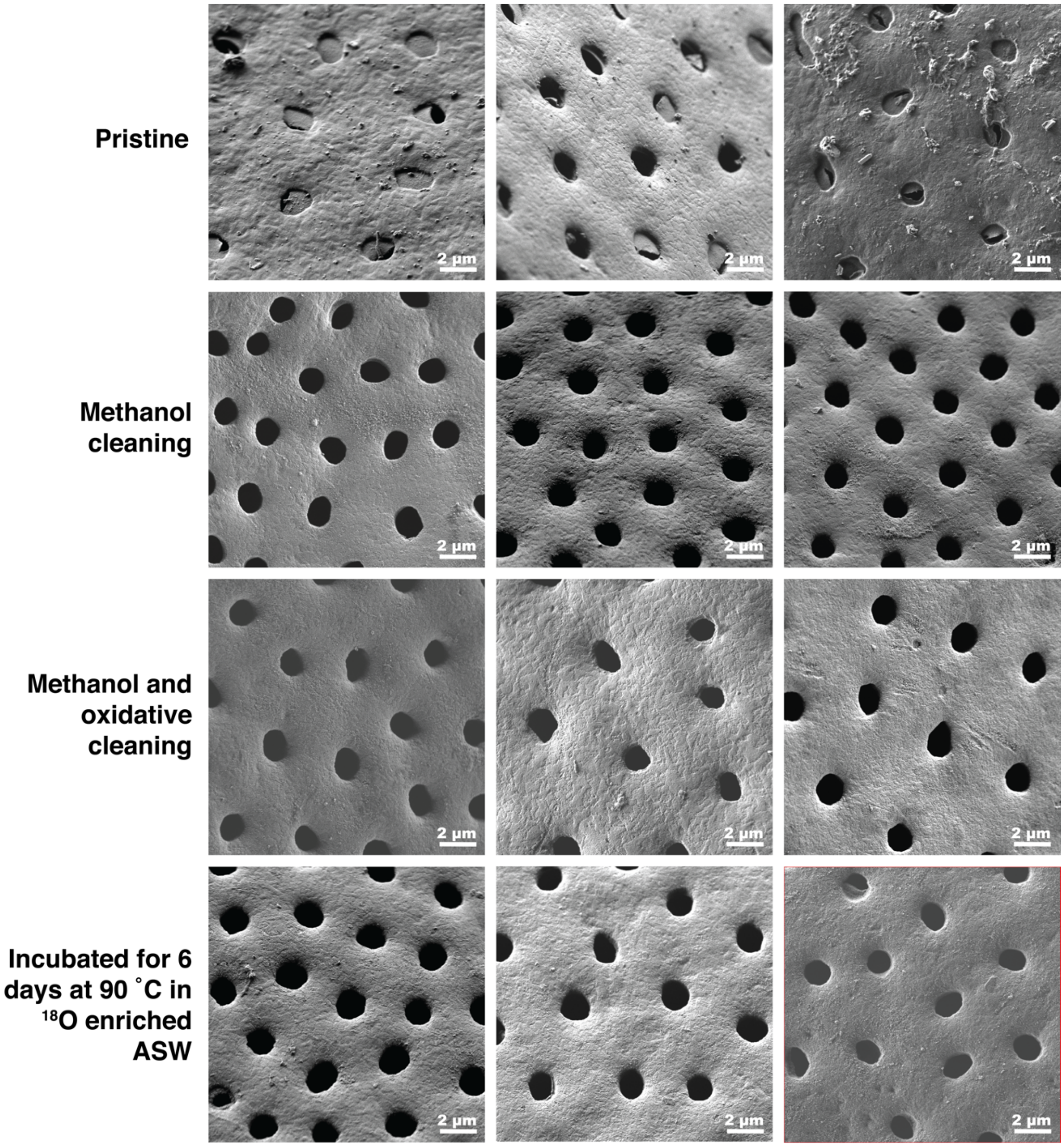

Supplementary Fig. 6 High magnification (5000×) SEM images of the surfaces of the penultimate chamber of *H. germanica* comparing tests that are pristine (1st row), cleaned only with methanol (2nd row), cleaned with methanol and an oxidative treatment (3rd row) to tests that were incubated for 6 days at 90 °C in artificial seawater (ASW) with a

$^{18}O/^{16}O$ ratio of 0.30 after methanol cleaning and an oxidative treatment. All images were taken at the same scale.

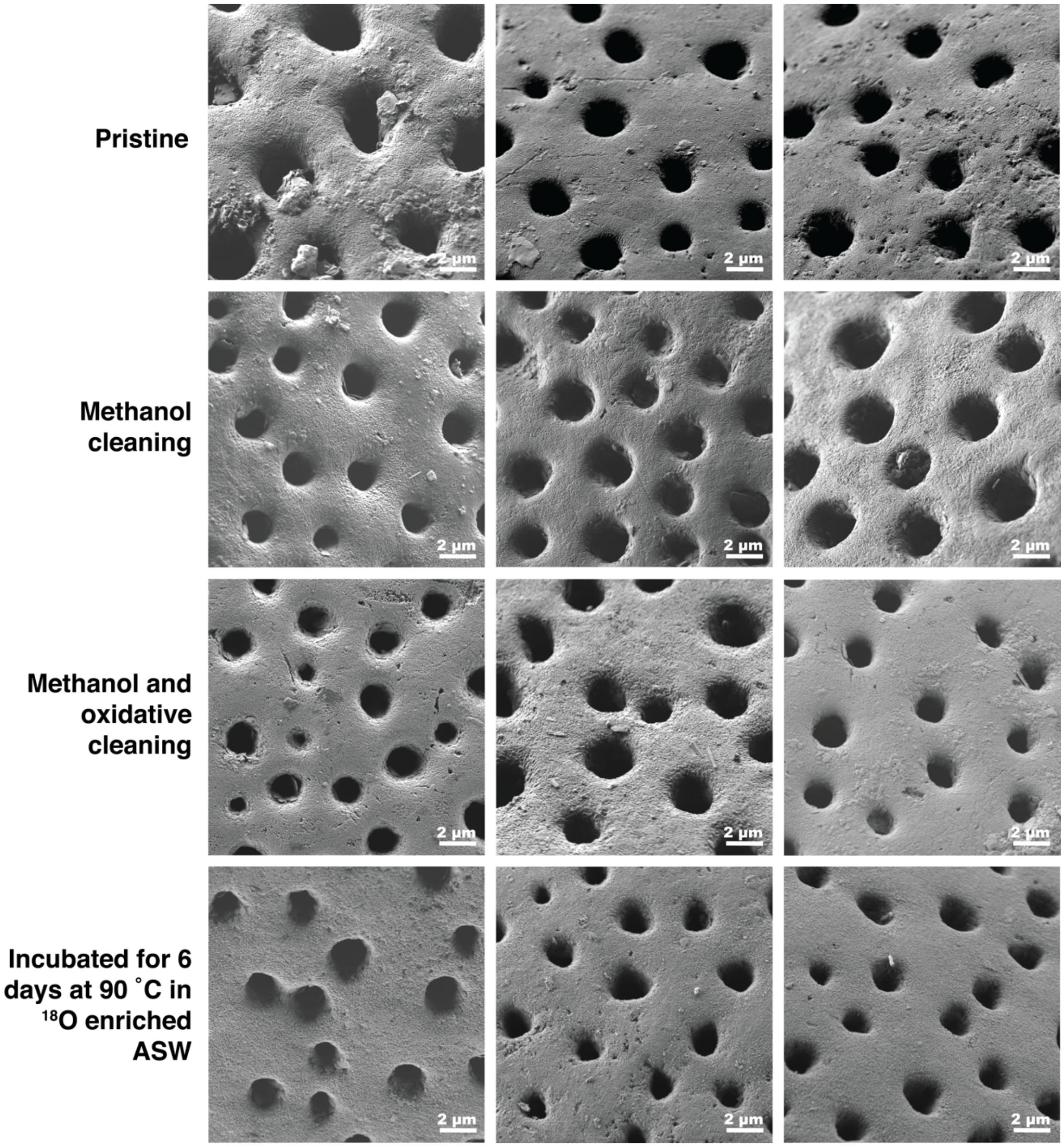

Supplementary Fig. 7 High magnification (5000×) SEM images of the surfaces of the penultimate chamber of *A. lessonii* comparing tests that are pristine (1st row), cleaned only with methanol (2nd row), cleaned with methanol and an oxidative treatment (3rd row) to tests that were incubated for 6 days at 90 °C in artificial seawater (ASW) with a

$^{18}O/^{16}O$ ratio of 0.30 after methanol cleaning and an oxidative treatment. All images were taken at the same scale.

Foram $\delta^{18}O$ (‰ VSMOW)			
**Species**	*Ammonia sp.*	*H. germanica*	*A. lessonii*
	508	722	782
	614	874	741
	499	761	726
	518	959	691
	472	687	674
	627	716	677
	483	743	747
	521	758	738
	507	705	676
	543	768	694
			657
			705
**Average**	529	769	709
**Standard Deviation**	49	80	36
**Variance**	2437	6398	1288

Supplementary Table 1 Summary table of the bulk oxygen isotope measurements of foraminifera tests that were incubated for 6 days at 90 °C in artificial seawater with a $^{18}O/^{16}O$ ratio of 0.30. Each measurement consists of 70–100 µg of foraminifera tests. The average, standard deviation and variance are reported for each species.

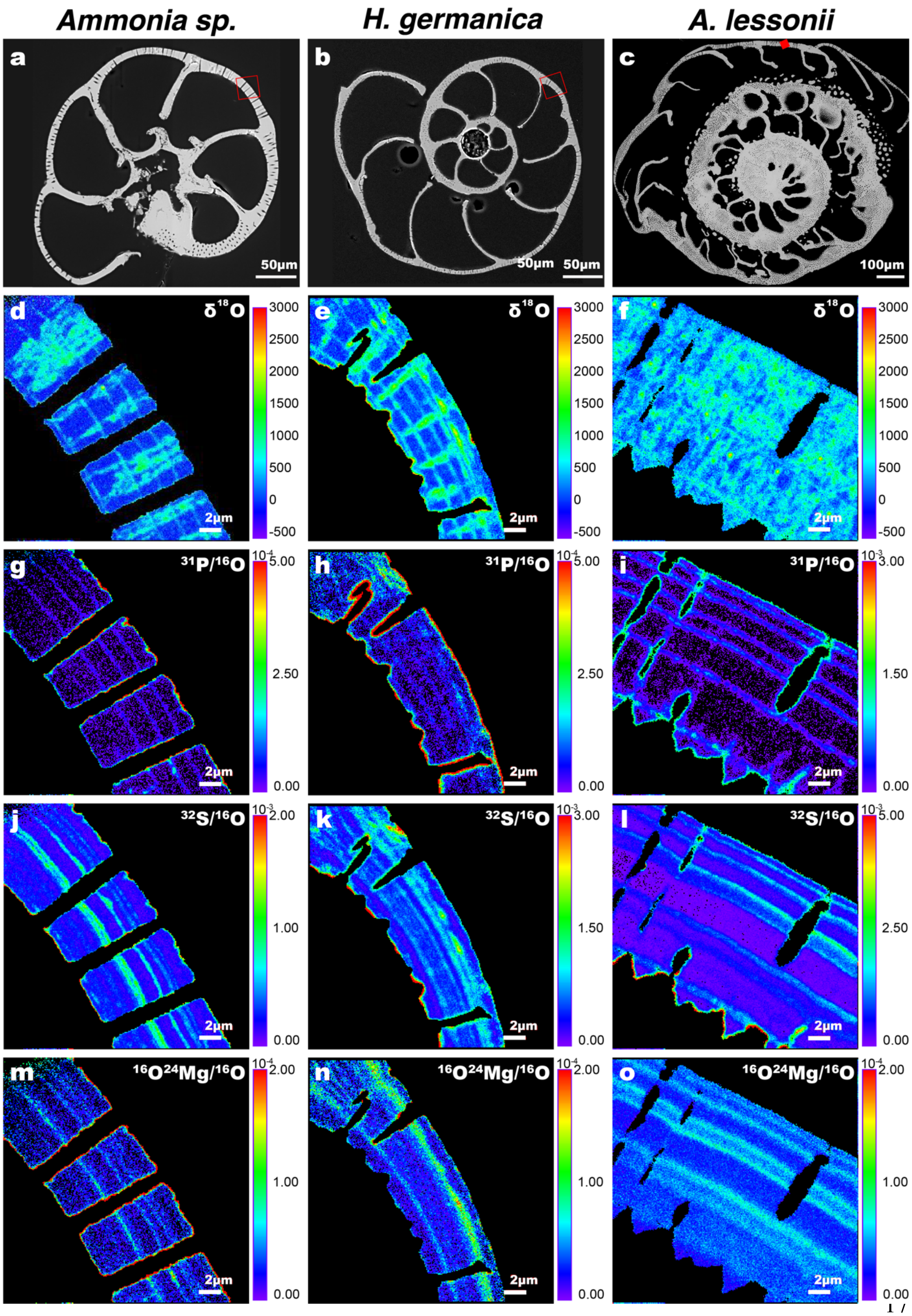
Ammonia sp.
H. germanica
A. lessonii
a
b
c
50μm
50μm
50μm
100μm
d
e
f
δ18O
3000
2500
2000
1500
1000
500
0
-500
2μm
g
h
i
31P/16O
10-4 5.00
2.50
0.00
10-3 3.00
1.50
j
k
l
32S/16O
10-3 2.00
1.00
10-3 3.00
1.50
10-3 5.00
2.50
m
n
o
16O24Mg/16O
10-4 2.00
1.00
0.00

Supplementary Fig. 8 NanoSIMS images of *Ammonia sp., A. lessonii* and *H. germanica* tests after incubation for 6 days at 90 °C in artificial seawater (ASW) with a $^{18}O/^{16}O$ ratio of 0.30. (a-c) shows SEM images of polished tests with the red square indicating the location of the NanoSIMS images in rows 2 to 5 that show surfaces parallel to pore axes. (d-f): $\delta^{18}O$. (g-i): $^{31}P/^{16}O$ ratio. (j-l): $^{32}S/^{16}O$ ratio. (m-o): $^{24}Mg^{16}O/^{16}O$ ratio. In all three species it is clear that ^{18}O-enrichment is best correlated with bands of P, as bands of S and Mg are thicker and more numerous than the bands of P. Additionally, the number of ^{18}O-enrichment bands correspond to the number of organic linings expected from the position of the chamber in question relative to the total number of chambers.

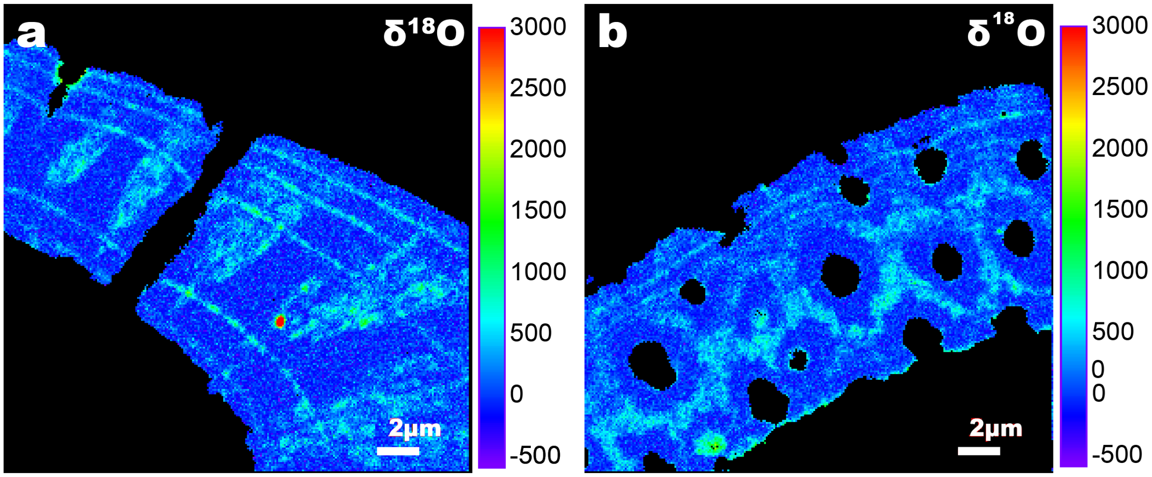

Supplementary Fig. 9 NanoSIMS images of an *Ammonia sp.* test after incubation for 4 hours at 90 °C in artificial seawater with a $^{18}O/^{16}O$ ratio of 0.30 after methanol cleaning and an oxidative treatment. Tests incubated for only 4 hours show the same heterogenous spatial pattern of ^{18}O-enrichment as experiments lasting 6 days, but with correspondingly lower ^{18}O-enrichments.

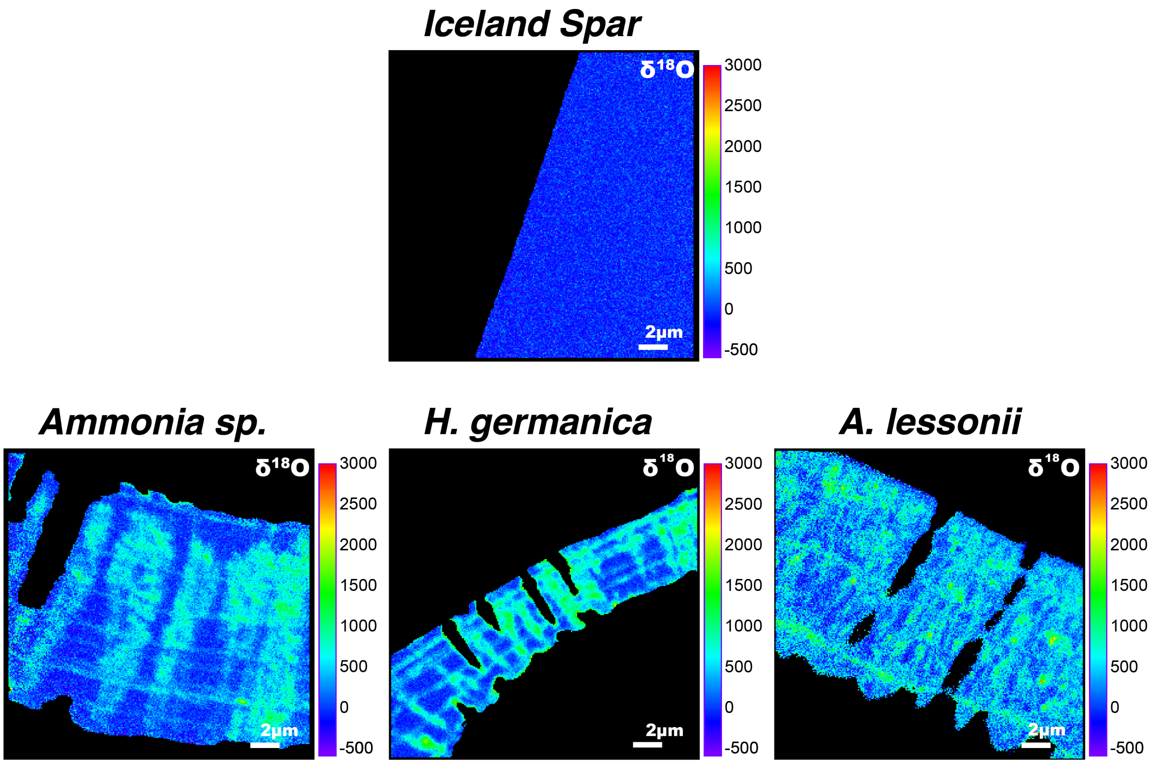

Supplementary Fig. 10 NanoSIMS $\delta^{18}O$-image of an Iceland spar crystal, in direct comparison with $\delta^{18}O$-images of *Ammonia sp., H. germanica* and *A. lessonii* tests after incubation for 6 days at 90 °C in artificial seawater with a $^{18}O/^{16}O$ ratio of 0.30 after methanol cleaning and an oxidative treatment. The interior of Iceland spar crystals did not become enriched in ^{18}O despite being incubated for the same time, in the same fluid, and at the same temperature as the benthic foraminifera tests.

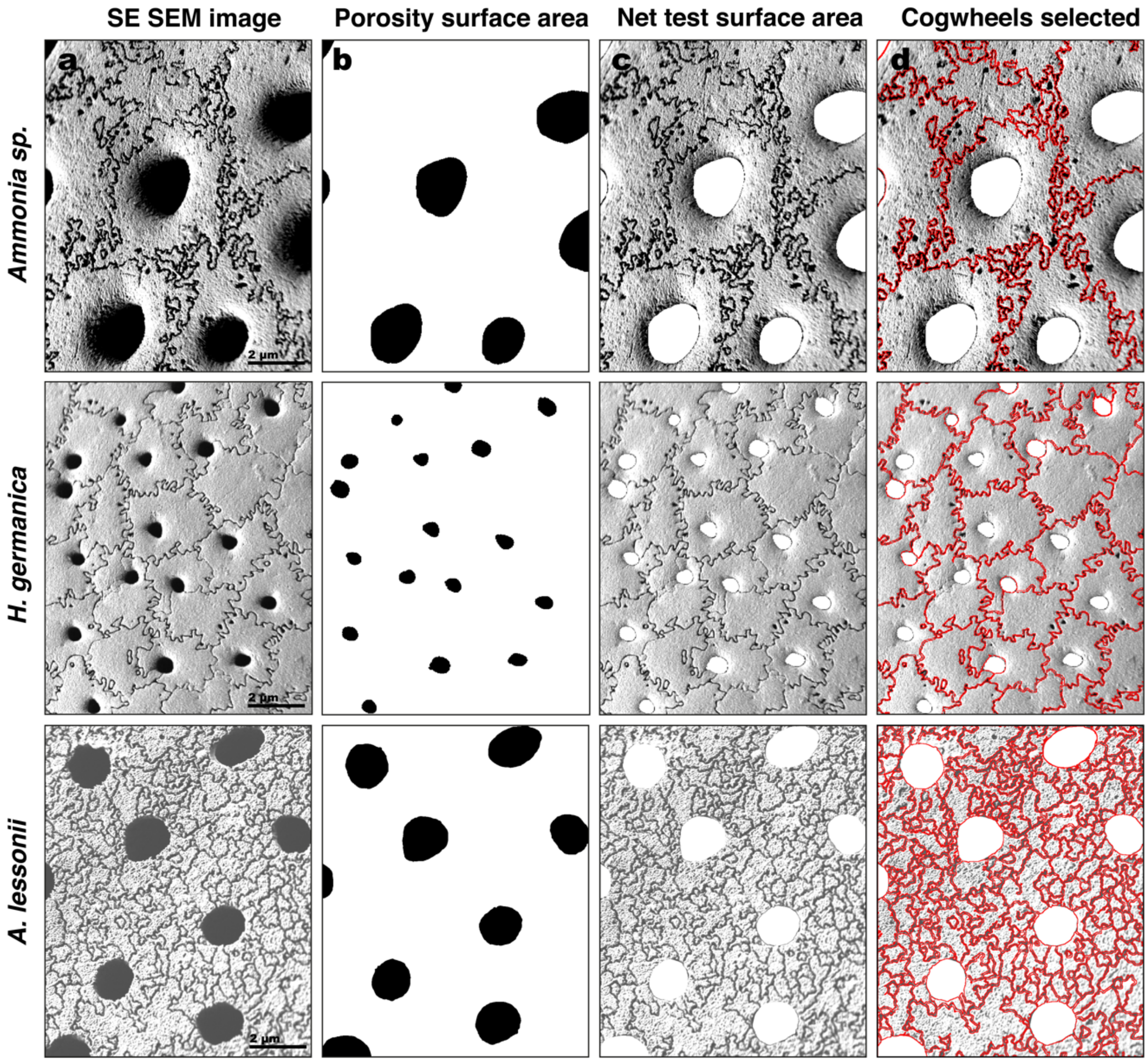

Supplementary Fig. 11 Images illustrating the 4 key steps of the ImageJ cogwheel quantification macro from van Dijk *et al.* [1] with an example from each of the three species used in this study: *Ammonia sp., H. germanica*, and *A. lessonii*. (A) The raw SEM images of external test surface areas were imported into ImageJ, and the images cropped such that only areas in sharp focus were used. (B) Gray value thresholding was used to select the pore spaces and the paintbrush tool was used to remove any pixels that do not form part of the pore surface area. (C) The net test surface area was

obtained by removing the pores from the cropped SEM image. (D) All cogwheels interfaces were registered and their total length measured.

Species	Image area dimensions	Porosity surface area (μm^2)	Net test surface area (μm^2)	Number of cogwheels	Total cogwheel interface length (µm)	Cogwheel interface density (µm per μm^2)
Ammonia sp.	21.79 um x 10.85 µm	24.59	211.83	95	381.40	1.80
Ammonia sp.	10.37 um x 11.33 µm	7.93	109.56	70	236.09	2.15
Ammonia sp.	15.23 um x 9.94 µm	25.26	126.13	49	180.95	1.43
					**Av. ± (1σ):**	**1.80 ± 0.36**
H. germanica	22.22 um x 14.91 µm	13.05	318.25	155	603.12	1.90
H. germanica	19.31 um x 13.19 µm	10.87	243.83	108	425.62	1.75
H. germanica	13.63 um x 11.24 µm	6.75	146.45	69	251.01	1.71
					**Av. ± (1σ):**	**1.78 ± 0.10**
A. lessonii	9.30 um x 11.57 µm	13.72	93.88	324	349.01	3.72
A. lessonii	10.90 um x 10.36 µm	17.14	95.78	282	322.17	3.36
A. lessonii	11.37 um x 10.36 µm	15.45	102.34	284	357.43	3.49
					**Av. ± (1σ):**	**3.52 ± 0.18**

Supplementary Table 2 Cogwheel interface density quantification parameters calculated using ImageJ for three foraminifera tests of *Ammonia sp., H. germanica,* and *A. lessonii* obtained by the procedure described in van Dijk *et al.* [1]. The net surface area was calculated by subtracting the area of the pores from the total area of the frame. The total cogwheel interface length (µm) is the total

length of all cogwheel interfaces excluding pore-calcite interfaces and pixels along the image frame; cf. Supplementary Fig. 11.

Cogwheel interface density (µm per µm^2) is calculated as the ratio of the total cogwheel interface length to the net test surface area.

The average cogwheel surface density for each species is provided ± one standard deviation.

Species	Test surface area (μm^2)	Maximum diameter	Number of chambers	Diagenesis resistance ratio
Ammonia sp.	35447	403	16	2215
Ammonia sp.	18967	364	13	1459
Ammonia sp.	25148	362	13	1934
Ammonia sp.	33922	423	15	2261
Ammonia sp.	20593	344	12	1716
Ammonia sp.	21661	368	13	1666
Ammonia sp.	27920	365	14	1994
Ammonia sp.	28960	404	15	1931
			**Av. ± (1σ):**	**1897 ± 274**
H. germanica	5428	214	9	603
H. germanica	14122	269	13	1086
H. germanica	10896	258	13	838
H. germanica	7320	212	10	732
H. germanica	16778	434	15	1119
H. germanica	15619	353	16	976
H. germanica	14190	333	14	1014
H. germanica	17168	393	18	954
			**Av. ± (1σ):**	**915 ± 178**

Supplementary Table 3 Parameters used to calculate the diagenesis resistance ratio, which is the ratio of the maximum cross-sectional test area (μm^2) to the number of chambers for *Ammonia sp.* and *H. germanica.* The average diagenesis resistance factor for each species is provided ± one standard deviation.

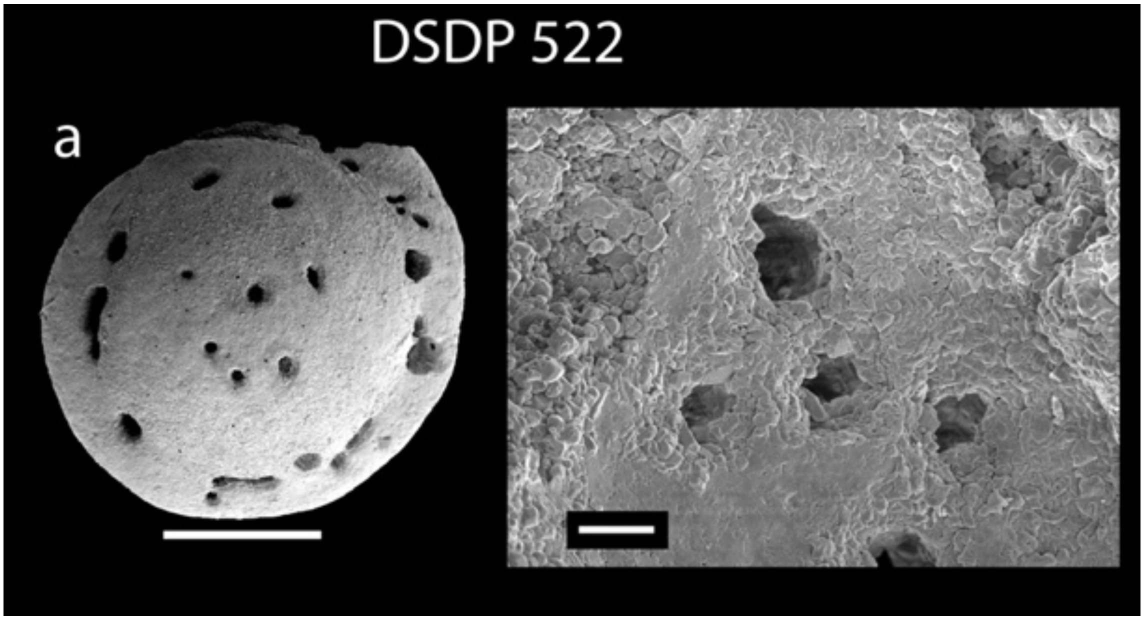

Supplementary Fig. 12 SEM image of the spiral side and high-magnification view of the wall texture of benthic foraminifera *Cibicidoides havanensis* from DSDP Site 522 (Walvis Ridge). Note the etching of pores and/or secondary calcite deposits on the surface. Scale bars are 100 μm (left side) and 10 μm (right side). Modified from Sexton *et al.* [2].

SI References

1. van Dijk, I. Van, Raitzsch, M., Brummer, G.-J. A. & Bijma, J. Novel Method to Image and Quantify Cogwheel Structures in Foraminiferal Shells. *Front. Ecol. Evol.* **8:567231**, 1–13 (2020).
2. Sexton, P. F., Wilson, P. A. & Norris, R. D. Testing the Cenozoic multisite composite δ18O and δ13C curves: New monospecific Eocene records from a single locality, Demerara Rise (Ocean Drilling Program Leg 207). *Paleoceanography* **21**, 1–17 (2006).
