# Supplementary material for: Fast and pervasive diagenetic isotope exchange in foraminifera tests is species-dependent
Source: Nat Commun. 2022 Jan 10;13:113. doi: 10.1038/s41467-021-27782-8 (PMC8748890; doi:10.1038/s41467-021-27782-8)
Supplement: Supplementary file 3 — Supplementary Software [file 41467_2021_27782_MOESM3_ESM.zip › Additional Supplementary Files/Sample images for Supplementary Software.pdf]

Supplementary Software for:

Fast and pervasive diagenetic isotope exchange in foraminifera tests is species-dependent

Deyanira Cisneros-Lazaro[1*], Arthur Adams[1], Jinming Guo[1], Sylvain Bernard[2], Lukas P. Baumgartner[3], Damien Daval[4], Alain Baronnet[5], Olivier Grauby[5], Torsten Vennemann[6], Jarosław Stolarski[7], Stéphane Escrig[1], Anders Meibom[1,3*]

* deyanira.cisneroslazaro@epfl.ch, +41779494386 , anders.meibom@epfl.ch +41216938014; EPFL ENAC IIE LGB, GR C2 524 (GR building), Station 2, CH-1015 Lausanne

ImageJ script for foraminifera test area and diameter analysis

This is a modified version of the “Cogwheel and pore analysis” ImageJ macro provided in van Dijk *et al.* [1] and is suitable for quantifying the area and diameter of foraminifera tests that have been embedded in epoxy and polished down to approximately the widest part of the test (using diamond polishing pastes with a final grain size of 0.25 µm) and imaged using backscattered electrons (BSE) SEM.

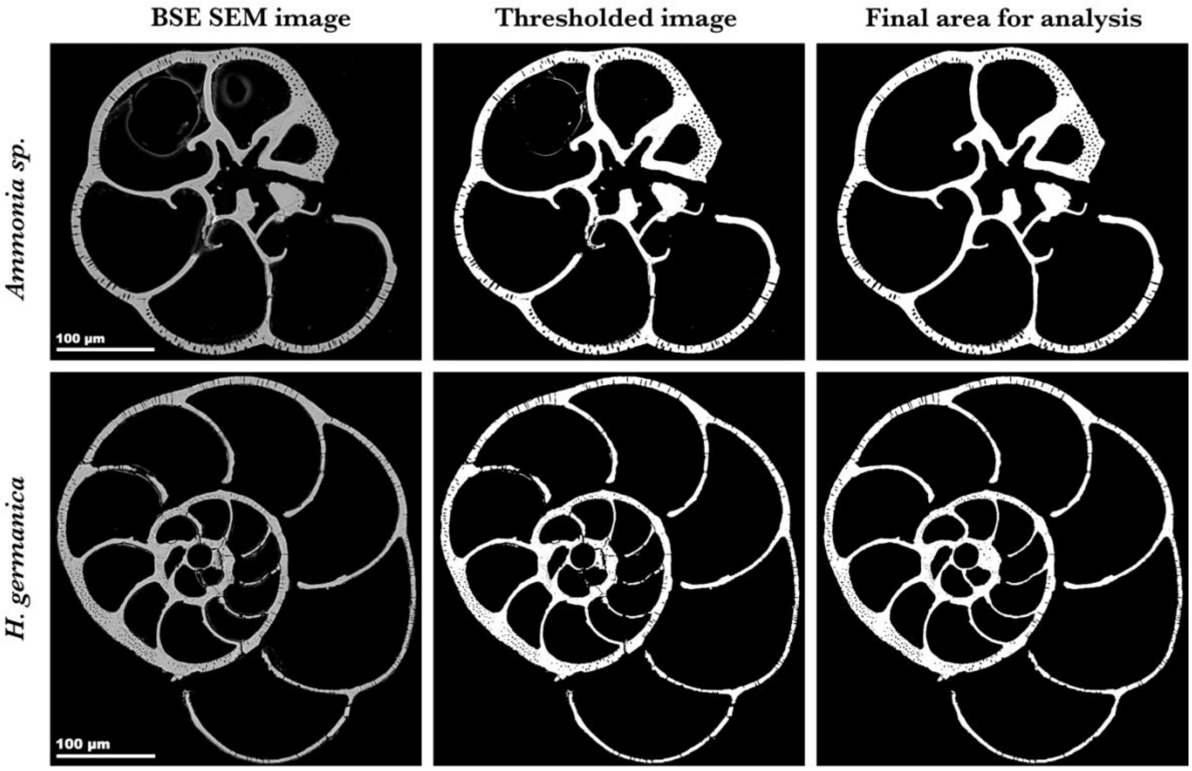

Script

```
macro "Test area analysis"

{

//Image preparation

//Set the scale

run("Set Scale...");      // when scale is the same for all images, change this line to: run("Set Scale...", "distance=xxx known=xxx pixel=1 unit=um");run("Set Scale...");

run("8-bit");      //convert image to grey scale values if not already done

run("Select All");
```

```
setTool("rectangle");
title = "Adjust selection ";
msg = "Adjust the selection frame to exclude e.g. text or scale bar if necessary, and click OK";
waitForUser(title, msg);
run("Duplicate...", "title=[Test selection]");

//Test selection
//Adjusting the threshold
run("Threshold...");
title = "Adjust threshold";
msg = "Adjust the threshold if necessary, by selecting 'Set', and click OK in this window";
waitForUser(title, msg);
getThreshold(lower, upper);
if (lower==-1)
exit("Threshold was not set");
run("Convert to Mask");
//remove dust/small particles from selection
run("Despeckle");
title = "Manual removal";
msg = "Any leftover unwanted selections can be removed using the Paintbrush tool. Press OK when finished";
waitForUser(title, msg);
run("Set Measurements...","area feret's");
//  Feret's diameter can be used to calculate the maximum diameter of the test, provided all parts of the test are joined together by at least one pixel.
run("Analyze Particles...", "size=0.01-Infinity show=Outlines display clear summarize");
title = "Export test area and diameter data";
```

```
msg = "Save results";
waitForUser(title, msg);


selectWindow("Drawing of Test selection");
run("Close" );
selectWindow("Summary");
run("Close" );
}
```

Supplementary Software References

1. van Dijk, I. Van, Raitzsch, M., Brummer, G.-J. A. & Bijma, J. Novel Method to Image and Quantify Cogwheel Structures in Foraminiferal Shells. *Front. Ecol. Evol.* **8:567231**, 1–13 (2020).
